# Subcutaneous axillary primary epithelioid hemangioendothelioma: report of a rare case

**DOI:** 10.1186/s40792-022-01521-7

**Published:** 2022-09-09

**Authors:** Takayoshi Niwa, Takaaki Konishi, Asako Sasahara, Ayaka Sato, Arisa Morizono, Mayumi Harada, Kotoe Nishioka, Osamu Fukuoka, Naohiro Makise, Yuki Saito, Mizuo Ando, Takako Yoshimoto, Takeshi Shikama, Satoshi Yamashita, Masahiko Tanabe, Yasuyuki Seto

**Affiliations:** 1 Department of Breast and Endocrine Surgery, Graduate School of Medicine, The University of Tokyo, 7-3-1 Hongo, Bunkyo-Ku, Tokyo, 113-8655 Japan; 2 Department of Otorhinolaryngology and Head and Neck Surgery, Faculty of Medicine, The University of Tokyo, 7-3-1 Hongo, Bunkyo-Ku, Tokyo, 113-8655 Japan; 3 Department of Pathology, Graduate School of Medicine, The University of Tokyo, 7-3-1 Hongo, Bunkyo-Ku, Tokyo, 113-8655 Japan; 4 Division of Surgical Pathology, Chiba Cancer Center, 666-2 Nitona-Cho, Chuo-Ku, Chiba-Shi, Chiba, 260-8717 Japan; 5 Department of Otolaryngology-Head and Neck Surgery, Faculty of Medicine, Dentistry and Pharmaceutical Sciences, Okayama University, Japan, 2-5-1 Shikata-Cho, Kita-Ku, Okayama, 700-8558 Japan; 6 Department of Gastrointestinal Surgery, Graduate School of Medicine, The University of Tokyo, 7-3-1 Hongo, Bunkyo-Ku, Tokyo, 113-8655 Japan

**Keywords:** Epithelioid hemangioendothelioma, Papillary thyroid cancer, Axillary tumor

## Abstract

**Background:**

Epithelioid hemangioendothelioma (EHE) is a rare and slow-growing malignant vascular neoplasm composed of epithelioid endothelial cells within a distinctive myxohyaline stroma. It most commonly involves somatic soft tissue, lungs, liver and bone. Herein, we describe a case of EHE arising in the axillary region.

**Case presentation:**

A 61-year-old man was under observation for multiple hepatic hemangiomas. Fluorodeoxyglucose–positron emission tomography/computed tomography showed specific uptake in a right axillary tumor. The patient was referred to our department for further investigation of the axillary tumor. An elastic-soft and poorly mobile tumor was palpable in the right axilla. Contrast-enhanced computed tomography showed a right axillary tumor and enlarged hepatic hemangiomas. In addition, multiple nodules in both lungs, a left renal angiomyolipoma, and left adrenal adenoma were revealed. Ultrasonography showed masses in both lobes of the thyroid gland, and a 30-mm lobulated hypoechoic mass in the axilla with well-defined and rough borders, showing internal heterogeneity. Fine-needle aspiration cytology was performed on the thyroid and axillary tumors: the thyroid tumor was class V, raising suspicion of papillary thyroid cancer (PTC); the left superior internal jugular node was class V, raising suspicion of metastasis of PTC; and the axillary tumor was class III, raising suspicion of a mesenchymal tumor with few epithelioid cells. The multiple lung nodules were diagnosed as metastatic tumors derived from thyroid cancer. We diagnosed these diseases as PTC of T1b(m)N1bM1(lung) Stage IVB and a right axillary tumor of unclear origin. However, it was assumed to be a primary mesenchymal tumor or a lymph node metastasis from lung cancer or occult breast cancer. We performed total thyroidectomy, left cervical lymph node dissection, and right axillary tumor excision. Histopathologic examination revealed the thyroid tumor as a PTC and the axillary tumor as an EHE. The EHE showed nuclear atypia, necrosis and high mitotic figures. Hence, it was considered to be a high-risk EHE.

**Conclusions:**

We experienced a rare primary subcutaneous axillary EHE with metastatic thyroid cancer in the lung. Since our case was classified as a high-risk EHE, a close follow-up would be appropriate.

## Background

Weiss and Enzinger first reported epithelioid hemangioendothelioma (EHE) in 1982 as a rare vascular endothelial neoplasm arising in soft tissues [1]. EHE had previously been regarded as an intermediary malignancy but was reclassified as a malignant tumor in the 2002 World Health Organization classification [2]. In recent years, EHE has been categorized as low grade or intermediate grade according to histological atypia [3]. The most common sites of EHE are superficial or deep soft tissues, followed by organs, such as bone, lungs and liver [4]. EHE can also involve the gingiva, mediastinum, and thyroid gland [5–7]. Histologically, the tumor cells are round or spindle-shaped with eosinophilic cytoplasm and round nuclei, presenting a so-called epithelioid cell-like appearance. The incidence of EHE is reportedly low, at just one in a million [8]. Most EHEs are relatively indolent, but their malignant potential varies among cases [9, 10]. Herein we describe a patient with axillary EHE as a comorbidity of papillary thyroid cancer (PTC) and hepatic hemangioma.

## Case presentation

A 61-year-old man had been referred to the Department of Hepatobiliary and Pancreatic Surgery of our hospital for multiple hepatic giant hemangiomas with no significant progression, 5 years before his first visit to our department. The hepatic hemangioma was diagnosed at age 52 during a routine physical examination. Computed tomography (CT), performed in June 20XX, showed newly emerging lesions, such as multiple nodules in both lungs, a right axillary tumor, and the already-known hepatic lesion (multiple enlarged hepatic hemangiomas). He was referred to our department for further examination of the axillary tumor.

**Fig. 1:**
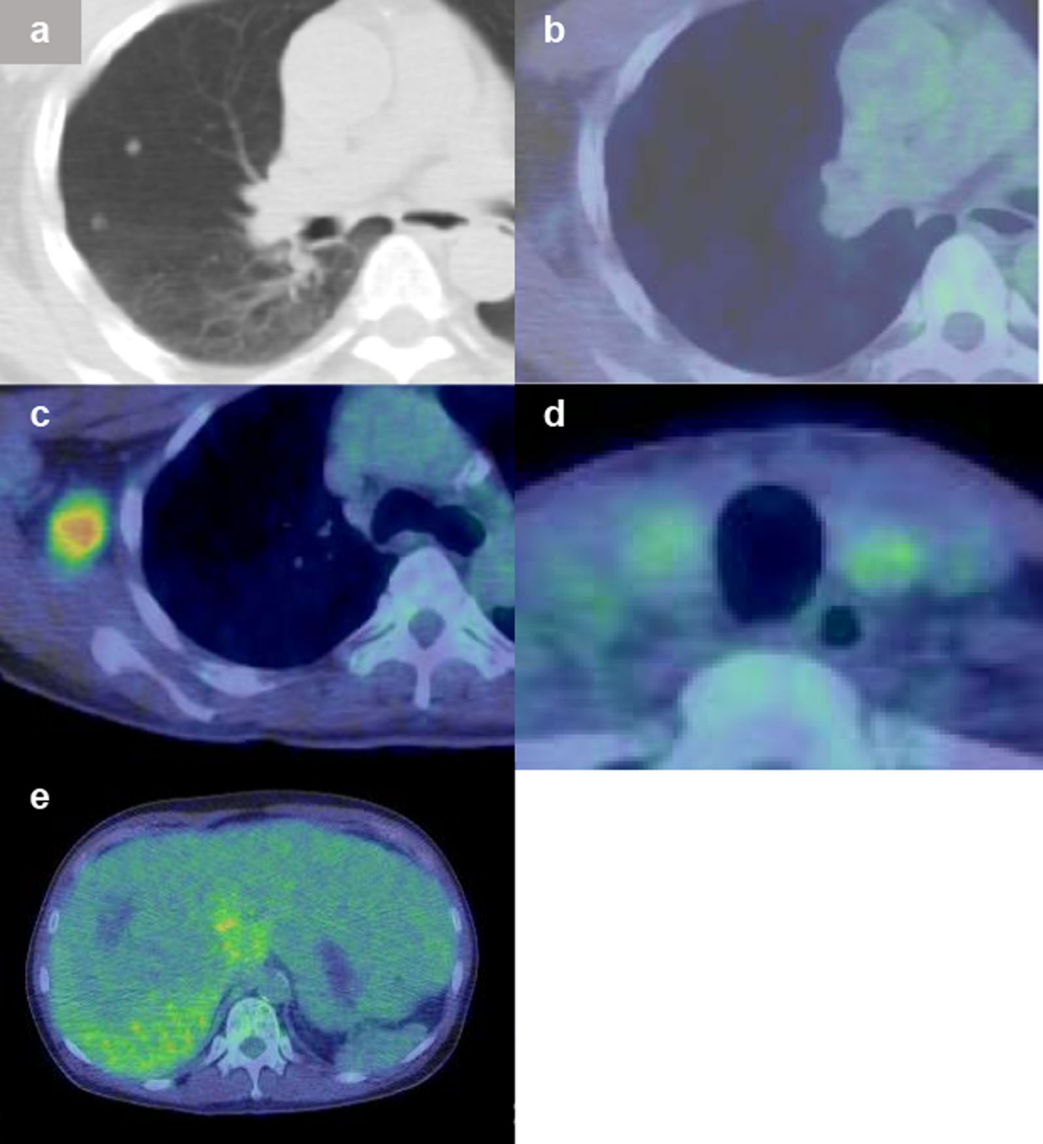
Fluorodeoxyglucose–positron emission tomography/computed tomography findings. **a**, **b** No specific uptake in multiple pulmonary nodules. **c** Right axillary tumor showed an accumulation with a standardized uptake value maximum of 5.1. **d** Both thyroid lobes showed only faint accumulation. **e** Decreased FDG accumulation consistent with a hepatic tumor was observed

Soluble interleukin-2 receptor (391 U/mL) and carcinoembryonic antigen (2.6 ng/dL) were within their normal ranges, while thyroglobulin was high at 226 ng/mL. Fluorodeoxyglucose–positron emission tomography (FDG–PET)/CT images were obtained in July of the same year. Whereas the thyroid gland showed faint FDG accumulation and multiple lung nodules showed no accumulation, FDG accumulation with a standardized uptake value maximum of 5.1 was observed in the right axillary tumor (Fig. 1a–d). A decrease in FDG accumulation in the liver, consistent with hepatic lesions was observed (Fig. 1e).

**Fig. 2:**
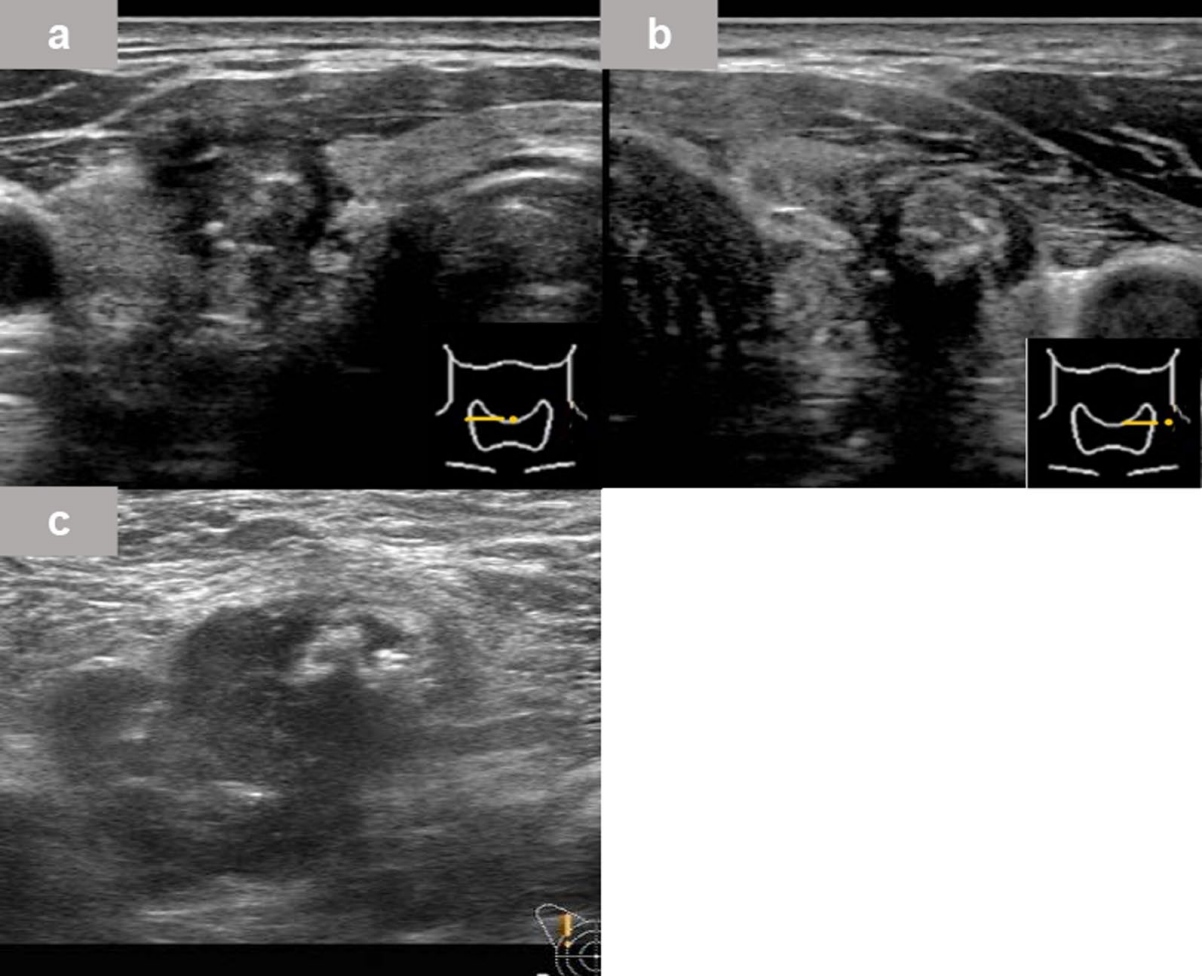
Ultrasonography findings. **a**, **b** Irregularly shaped, ill-defined, hypoechoic mass was seen in the right (**a**) and left (**b**) lobes of the thyroid gland. There were hyperechoic spots with an acoustic shadow. **c** Right axillary tumor was a hypoechoic tumor. It had a well-defined and rough border. It showed internal heterogeneity and its size was 30 mm. There was no obvious enlargement of surrounding lymph nodes

Cervical ultrasonography showed masses in both lobes of the thyroid gland. Both tumors were irregularly shaped and hypoechoic with indistinct borders, and the mass in the right lobe was suspected of having capsular invasion (Fig. 2a, b). The right cervical lymph nodes were swollen, suggesting metastasis of the thyroid tumor. A hypoechoic mass was found in the right axilla (Fig. 2c). It was lobulated with well-defined and rough borders and showed internal heterogeneity. The majority of the mass was hypoechoic with some hyperechoic areas. The mass was 30 mm in diameter, and there was no apparent enlargement of the other axillary lymph nodes. In bilateral breast ultrasonography, there were no abnormal findings in his right breast.

**Fig. 3:**
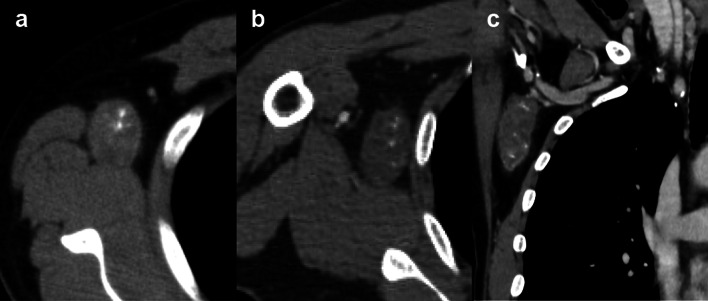
Computed tomography (CT) findings of the axillary tumor. **a** CT showed a mass with a high absorption area. **b** On contrast-enhanced CT, the tumor showed poor contrast with neither chest muscle nor chest wall invasion. **c** In the frontal view, there was no obvious invasion of the subclavian vein. The subclavian artery was intact, but the dorsal thoracic artery could not be identified

**Fig. 4:**
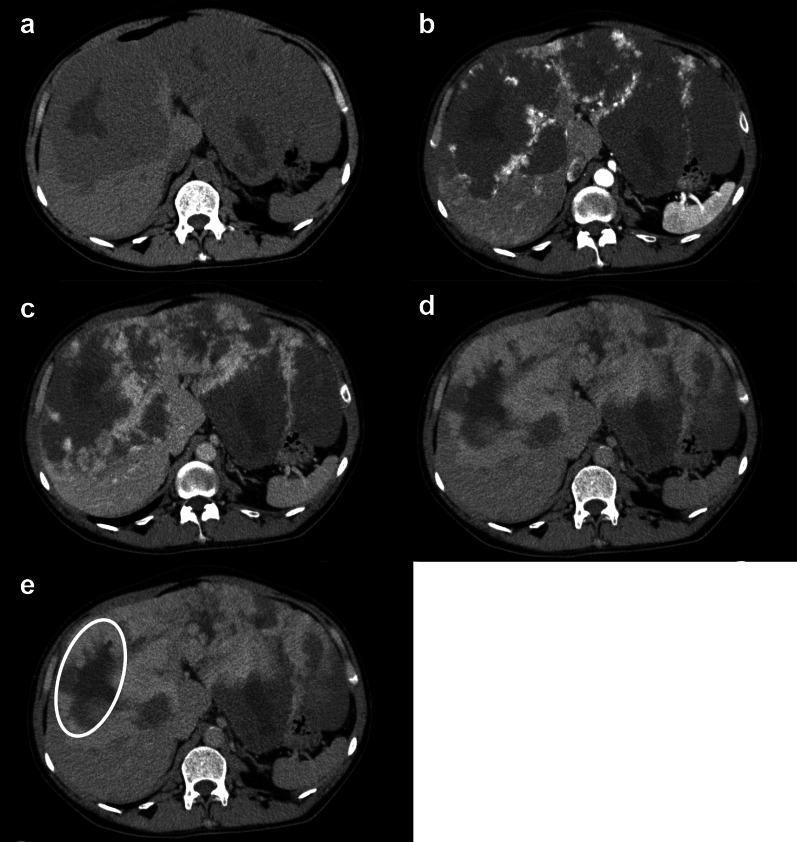
Cavernous hemangioma of the liver. **a** Unenhanced series. Native computed tomography showed multicentric, low density, well-defined tumors with central hypodensity. **b** In the arterial phase, early staining of the peripheral part of the tumor was observed. **c–e** Portal venous (parenchymal) and delay phase showed extension of the contrast effect to the center. An area that was not enhanced was observed in the tumor (white circle)

Contrast-enhanced CT showed multiple nodules with calcification in both thyroid gland lobes. In the right axilla, there was an oval mass with a high-density area and well-defined borders. The contrast enhancement was poor, and there was no evidence of either muscle or chest wall invasion (Fig. 3a, b). In the frontal view, the subclavian artery was intact, but the dorsal thoracic artery could not be identified (Fig. 3c). Multiple nodules up to 7 mm were found in both lobes of the lung. The liver nodules demonstrated early staining in the arterial phase and the contrast effect spread to the center of the tumor in the portal phase, corresponding to a known hemangioma (Fig. 4a–e). Since these CT findings were typical of hepatic hemangiomas, we diagnosed liver nodules with hepatic cavernous hemangioma. FDG–PET/CT findings also supported that this space-occupying lesion (SOL) in the liver was not highly suspicious of being a malignant tumor (Fig. 1e). In addition, left renal angiomyolipoma, left adrenal adenoma, and a subcutaneous lipoma was detected.

Fine-needle aspiration cytology was performed on the thyroid tumors, cervical lymph nodes, and right axillary tumor: the right thyroid tumor was class IV, raising suspicion of PTC; the left thyroid tumor was class V, also raising suspicion of PTC; the right cervical lymph node was class II, benign; the left cervical lymph node was class V, raising suspicion of metastasis of PTC; and the right axillary tumor was class III. Cytological examination of the axillary tumor revealed epithelial-like cells with rhombic to pleomorphic sporophytes that were solitary and scattered, accompanied by a mass of mesenchymal tissue. Immunohistochemically, thyroid transcription factor-1 was negative.

The patient was thus diagnosed with PTC and an unspecified right axillary tumor. The presumptive histology of the axillary tumor was consistent with a mesenchymal tumor. The differential diagnosis of the axillary tumor included a lymph node metastasis from PTC and occult breast cancer.

The multiple lung nodules were clinically diagnosed as metastatic tumors derived from thyroid cancer. We diagnosed these diseases as PTC of T1b(m)N1bM1(lung) Stage IVB and a right axillary tumor.

The patient underwent total thyroidectomy, cervical lymph node dissection and axillary tumor resection. The axillary tumor showed no obvious direct invasion of the axillary arteriovenous system. However, direct invasion of the dorsal thoracic artery was observed, and the artery was ligated at the branch from the axillary artery and resected.

**Fig. 5:**
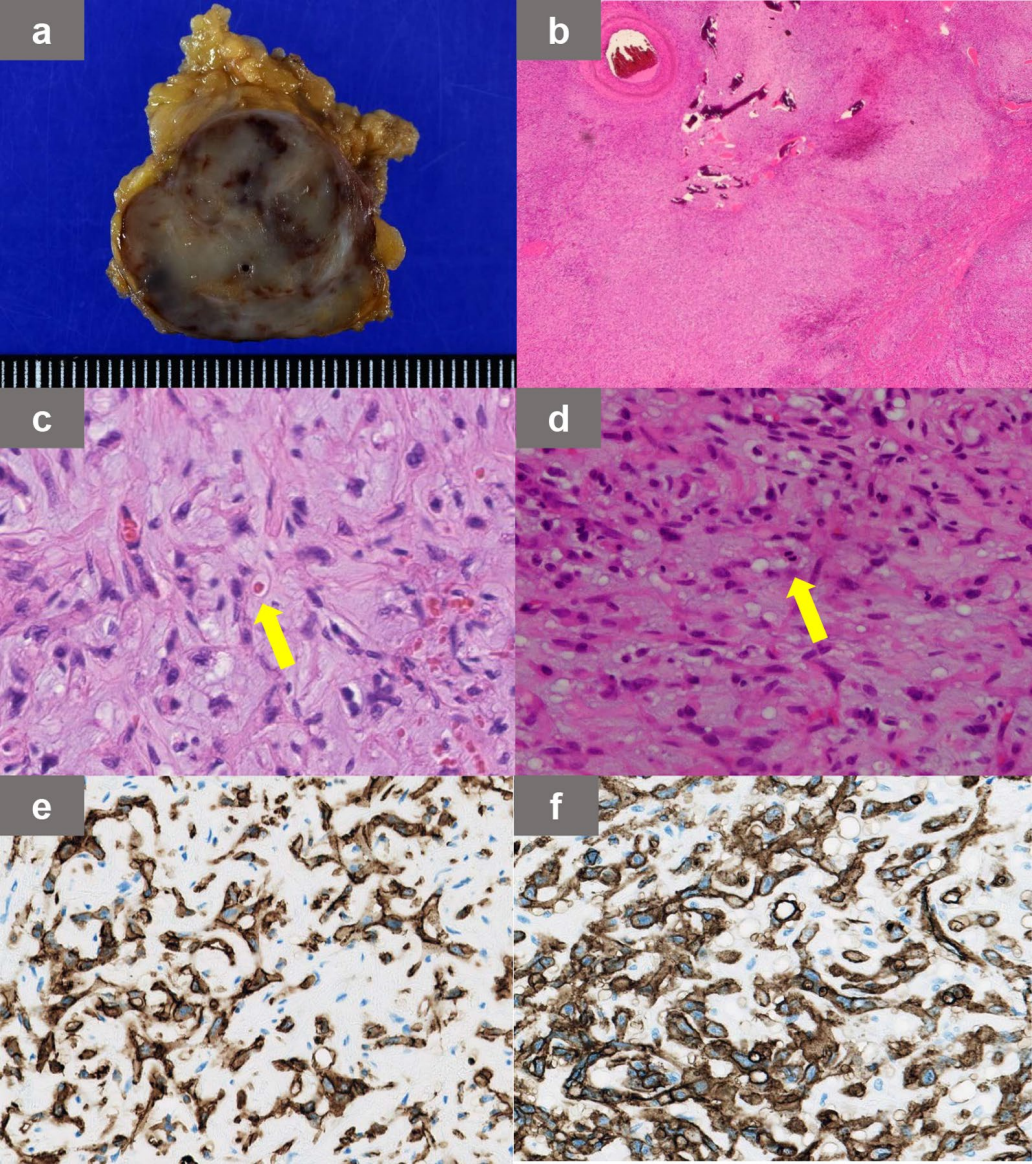
Histopathological findings of the axillary tumor. **a** Macroscopic findings of the axillary tumor. **b** Tumor was angiocentric. **c** Some tumor cells showed a primitive vascular cavity (arrow) that could contain red blood cells. **d** Nuclear atypia was marked, and mitotic figures (arrow) of about three cells/2 mm^2^ were observed. **e**, **f** Immunostaining showed tumor cells to be positive for the vascular markers, CD31 (**e**) and CD34 (**f**)

In postoperative pathology, multiple nodules were macroscopically observed in both lobes of the thyroid more than expected preoperatively. The pathological diagnosis was PTC. The axillary tumor was 45 mm in its longest diameter, and gross examination showed hemorrhage, calcification and necrosis within the tumor (Fig. 5a). Histologically, the tumor was angiocentric in origin and invaded surrounding soft tissue, adipose tissue, and nerve tissue in a lobulated manner with fibrous septa (Fig. 5b). The borders were relatively clear. The background was a myxohyaline substrate, and epithelioid to spindle-shaped cells proliferated in cord-like and vesicular patterns, some of which formed blood vessels. The individual tumor cells had mildly pleomorphic nuclei and eosinophilic cytoplasm and were observed to form primitive vascular spaces in which red blood cells were present (Fig. 5c). There was marked nuclear atypia in the cells, and mitotic nuclei were detected in three cells/2 mm^2^ (Fig. 5d). Immunostaining showed the axillary tumor to be positive for cluster of differentiation (CD) antigen 34, CD31 (Fig. 5e, f) and erythroblast transformation specific-related gene, while negative for cytokeratin AE1/3, epithelial membrane antigen, and thyroid transcription factor-1 (data not shown). In addition, distinct nucleoli, extensive necrosis, and sinusoidal vascular lumen formation, which were typical features of hemangiosarcoma, were not observed. Consequently, the tumor was diagnosed as EHE without containing lymphatic tissue. The large tumor size, nuclear atypia, and high mitotic activity indicated that our case should be classified as high-risk EHE.

Finally, the patient was diagnosed with PTC (pT3bN1bM1[lung] stage IVB) and primary axillary EHE. After surgery, we administered radioiodine therapy (^131^I 100 mCi, 3.7 GBq) twice for lung metastases of thyroid cancer, and the patient is currently receiving follow-up care. The hepatic hemangioma is also under follow-up in the Hepatobiliary and Pancreatic Surgery department. As for the EHE, the patient remains free of recurrence to date.

## Discussion

### Histopathological features and malignant potential of EHE

EHE is characterized by cords and nests of epithelioid cells in a myxohyaline stroma [11]. Intracytoplasmic vacuoles containing erythrocytes are also a distinctive feature. As observed in our case, two to four of these cells aggregate to form an elongated slit-like lumen, and the lumen contains erythrocytes. Immunostaining showed tumor cells to be positive for at least one of the vascular markers, that is, CD31, CD34, factor VIII-related antigen or Ulex europaeus agglutinin-1, while negative for epithelial cell membrane antigen [12, 13].

Prognostic stratification into a high-risk category is based on mitotic activity, nuclear atypia and tumor size [14]. In most cases, the mitotic count is very low. According to a previous report, more than three mitotic figures per 50 high power fields (assuming an area of 0.2 mm^2^) indicate a high-risk classification [15]. Therefore, our patient would be regarded as a high-risk case. Adjuvant systemic treatments nor periodical surveillance after operation have not been established in EHE. However, the latter might be clinically significant, because detecting distant metastases earlier would enable us to treat the disease before its progression.

The presence of marked nuclear atypia and high mitotic nuclei required differentiation from epithelioid angiosarcoma. Immunohistochemical staining of CAMTA1 can help the differentiation of EHE and epithelioid angiosarcoma [16]. Unfortunately, we could not perform CAMTA1 immunostaining due to the lack of suitable antibodies in our institution. However, the histopathological features of the presented case were quite different from that of epithelioid angiosarcoma; that is, the current case did not demonstrate distinct nucleoli, extensive necrosis or sinusoidal vascular lumen formation [17]. In addition, the number of atypical nuclei and mitotic figures in the present case were fewer than seen in epithelioid angiosarcoma. Therefore, we diagnosed the axillary tumor as EHE.

### Background diseases and EHE

In the present case, tumors were observed in multiple organs. Therefore, the association between EHE and the other lesions, including their differential diagnoses, needed to be discussed based on clinical and histological features.

The liver is one of the most common sites of EHE [4]. Dynamic CT findings of EHE show mainly a mild enhancement at the tumor margins in the late phase [18]. On the other hand, the typical findings of hepatic cavernous hemangioma are early staining with a density equal to that of the aorta in the arterial phase of dynamic CT, high absorption in the portal or late phase, and extension of the contrast effect to the center [19]. Once these findings are observed, additional examinations are usually unnecessary. In our present case, hepatic tumors were diagnosed as cavernous hemangioma, because the tumors demonstrated typical CT findings of hepatic hemangiomas, distinctly different from that of an EHE. In addition, FDG–PET/CT may be useful to differentiate benign from malignant vascular tumors. If the SOL in the liver had been malignant, an increase in FDG accumulation would have been observed as previously reported [20]. In our case, both in hypervascular and hypovascular areas, FDG accumulation was reduced over the entire extent of the liver tumors.

Moreover, another paper describing 402 patients with malignant hepatic EHE reported no patients with metastases to soft tissues [18]. Therefore, we ruled out the possibility that the axillary tumor was a lymph node metastasis originating from hepatic EHE. Since there was no development of the tumors during periodic follow-up in the Hepatobiliary and Pancreatic Surgery department, we diagnosed the SOL of the liver as a hepatic hemangioma without biopsy.

An axillary lymph node metastasis of PTC was considered a possible diagnosis for the axillary tumor. Indeed, a case of PTC with the manifestation of axillary lymph node metastasis has been reported [21]. Metastasis of PTC to axillary lymph nodes is very rare but should be considered. In the current case, although we did not perform a core needle biopsy of the axillary tumor because of the proximity of the axillary vein, the histological findings and immunostaining of the axillary tumor allowed us to rule out axillary lymph node metastasis of PTC. On the other hand, EHE can reportedly occur in the thyroid gland [7], but the thyroid lesion in our present case was diagnosed as a typical PTC, based on histopathological examination.

For the multiple pulmonary nodules, it was difficult to distinguish metastases originating from PTC or EHE based on CT findings alone; that is, both showed similar bilateral multiple nodular opacities [22]. In the present case, PTC was an advanced thyroid cancer with intra-glandular and cervical lymph node metastasis, which likely had lung metastasis. Moreover, the lung nodules demonstrated no accumulation in the FDG–PET/CT images, whereas the axillary tumor demonstrated apparent accumulation. Therefore, we considered the lung nodules were metastases from the thyroid cancer not the axillary EHE.

In addition to multiple hepatic hemangiomas, thyroid cancer and an axillary mass, the patient was also diagnosed with left renal angiomyolipoma, left adrenal adenoma and subcutaneous lipoma. Cowden syndrome is an autosomal dominant syndrome and is also known as a form of phosphatase and tensin homolog hamartoma tumor syndrome. There is a high risk of tumors in multiple organs, including skin, intestine, breast and thyroid. In addition to benign disease, breast and thyroid cancers are found in individuals with this syndrome [23, 24]. Our literature search yielded no reports describing an association between Cowden syndrome and EHE. However, although the clinical criteria for Cowden syndrome were not met, it remains possible that the multiple lesions in the present case might be related to an as-yet-unknown genetic predisposition.

## Conclusions

We experienced a rare case of primary subcutaneous axillary EHE presenting as a comorbidity of thyroid cancer. Histopathologically, this case had increased mitotic figures, nuclear atypia, coagulative tumor necrosis, and large tumor size, suggesting a high malignant potential. Intensive follow-up remains essential, along with treatment of the lung metastasis from thyroid cancer.

## Data Availability

All data supporting the conclusions of this article are included within the published article.

## Abbreviations

EHE: Epithelioid hemangioendothelioma
FDG: Fluorodeoxyglucose
PET: Positron emission tomography
CT: Computed tomography
SUV: Standardized uptake value
PTC: Papillary thyroid cancer
CD: Cluster of differentiation
SOL: Space-occupying lesion
